# Experienced Protective Factors While Growing up With Reading Disability

**DOI:** 10.1177/07319487241302309

**Published:** 2024-12-13

**Authors:** Tuija Aro, Mari-Lotta Rossi, Leena Paakkari, Minna Torppa

**Affiliations:** 1 University of Jyväskylä, Finland; 2 Niilo Mäki Institute, Finland

**Keywords:** reading disability, adulthood, experiences of protective factors

## Abstract

We studied lifelong factors that were experienced as protective by 48 Finnish adults (20–39 years) whose reading disability (RD) was diagnosed in childhood. Our aim was to gain a better understanding of the factors that can help individuals live with RD. We used semi-structured interviews and qualitative content analysis. Three main themes were identified: Factors Supporting Personal Development (four subthemes), Factors Supporting Social Well-being and Belonging (two subthemes), and Factors Supporting Learning (two subthemes). Our findings emphasize that in addition to an individual’s personal attributes and personal development, several protective factors can be identified from the acceptive and safe social environment, and social relationships. Besides, flexible and individual support for learning in school can make a difference. These findings can guide the planning and provision of the appropriate personal, social, and practical learning support, and they have implications for future studies on protective factors among individuals with RD.

Reading disability (RD) is one of the specific learning disorders (*Diagnostic and Statistical Manual of Mental Disorders, 5th ed.* [*DSM*-5]; American Psychiatric Association, 2013) that are among the most frequently diagnosed developmental disorders in childhood; RD affects about 7% of school-age children (Yang et al., 2022). Despite being commonly diagnosed in childhood, RD often persists, impairing reading and spelling skills even in adulthood (e.g., Eloranta et al., 2019). In addition to hampering the development of academic skills, RD may have adverse effects on an individual’s life course and psychosocial well-being (Hakkarainen et al., 2015; Ingesson, 2007; McLaughlin et al., 2014; Wilson et al., 2009). Although these negative adult-age outcomes have been known by researchers, surprisingly little research has focused on factors that could alleviate these adverse consequences or protect from them over the life course. In this study, we aimed to fill the gap in the knowledge on protective factors by interviewing adult participants grown-up with RD. We were interested in their lifelong experiences that they themselves report as being supportive, protective, or helping them to cope with their RD and its consequences.

## Long-Term Consequences of RDs

Quantitative studies among adolescents and adults have shown that RD is associated with lower educational attainment (T. Aro et al., 2019; Hakkarainen et al., 2015) and a lower income level (McLaughlin et al., 2014). Along with these, problems with mental health have been reported (T. Aro et al., 2019, 2024; Ghisi et al., 2016; Klassen et al., 2013; Wilson et al., 2009). Although we yet lack meta-analyses summarizing or aggregating the previous findings among adults with RD (except Klassen et al., 2013), it can be concluded that the quantitative studies have mainly focused on challenges and adverse outcomes, and thus, they have painted a rather negative picture of the adult-age outcomes of RD.

Furthermore, qualitative studies among adults with a history of RD have demonstrated that also somewhat more subtle problems in self-concept or self-esteem (Carawan et al., 2016; Hellendoorn & Ruijssenaars, 2000; Stampoltzis & Polychronopoulou, 2009), academic progress, opportunities, and choices, and negative school experiences more generally (Bacon & Bennett, 2013; Doikou-Avlidou, 2015; Stampoltzis & Polychronopoulou, 2009) and problems in work participation (for a review, see de Beer et al., 2022) and in social relations, and having negative emotional experiences (Doikou-Avlidou, 2015; Lithari, 2019; Stampoltzis & Polychronopoulou, 2009) are common among individuals with RD as they grow up. Similar findings on academic self-esteem and academic choices have been found among teenagers and young adults (Ingesson, 2007).

Despite the finding on negative long-term outcomes related to RD, it is known that some individuals with childhood RD do not face mental health or other negative consequences in adult age. Nevertheless, the pathways from having RD to experiencing—or not experiencing—educational or mental health problems later in life are not known. We assume that the more subtle negative experiences shown in the qualitative studies might be pivotal on the path to the often reported more severe difficulties related to education, income, and mental health (e.g., T. Aro et al., 2019; Ghisi et al., 2016; Hakkarainen et al., 2015; Klassen et al., 2013; McLaughlin et al., 2014). Furthermore, we assume that experienced protective factors related to these more subtle problems could be perceived as relevant by the individuals and may protect them from the negative outcomes. However, these subtle experiences are easily obscured in the analysis of large amounts of quantitative data. Conversely, using qualitative research methods allows us to gain in-depth insights of individuals’ experiences by capturing their lived experiences and complexities in them, and here, to explore the nuanced and subjective nature of experienced protective factors among adult individuals with RD.

## Qualitative Findings on Experienced Protective Factors

Despite the lack of research that specifically targets experiences of protective factors, some of the previous qualitative findings speak to this aim, suggesting possible supportive or protective elements in different domains. First, some studies have identified personal characteristics, such as perseverance and responsibility (e.g., Hellendoorn & Ruijssenaars, 2000), being experienced as useful when living with RD. Second, early diagnosis/recognition and acceptance of the disability (Doikou-Avlidou, 2015; Hellendoorn & Ruijssenaars, 2000; Lithari, 2019; Riddick et al., 1997; Stampoltzis & Polychronopoulou, 2009), and third, having a caring and understanding environment comprising parents, peers, friends, work colleagues, and teachers (Doikou-Avlidou, 2015; Hellendoorn & Ruijssenaars, 2000; Lithari, 2019; Macdonald, 2009; Nalavany et al., 2011; Stampoltzis & Polychronopoulou, 2009) have been noted as positive experiences by people with RD. Finding the right niche (McNulty, 2003) or achieving success (e.g., working with and validating one’s “strengths,” having a job that brings satisfaction, and engaging in physical activities or hobbies; Nalavany et al., 2011) has also been found to enhance an individual’s coping skills or quality of life.

However, the previous studies have been conducted with a variety of methods, making it difficult to draw conclusions, and many of them have used exclusive samples, mainly university students (Bacon & Bennett, 2013; Doikou-Avlidou, 2015; Lithari, 2019; Shaywitz et al., 2020; Stampoltzis & Polychronopoulou, 2009) or participants in specific occupations, such as nurses (Major & Tetley, 2019), doctors (Locke et al., 2017; Newlands et al., 2015), or teachers (Burns et al., 2013). Only a few studies have included individuals from various educational backgrounds (Hellendoorn & Ruijssenaars, 2000; McNulty, 2003; Nalavany et al., 2011), although it can be surmised that the findings among individuals with higher levels of education are not fully generalizable to the full population of people living with RD. For instance, those whose education ended after elementary school or who followed a vocational educational track may have different experiences and perceptions than those who continued pursuing their school career.

## The Present Study

Although the previous studies have focused on college or university education students, the interviews in this study were conducted among adults (20–39 years) with RD who had diverse educational backgrounds varying from no education after compulsory education to university degree. Moreover, none of the previous studies with participants from diverse educational backgrounds has been conducted in Finland, the context of this study. In Finland, learning difficulties are commonly detected by the classroom teacher or special education teacher. Each school has its own special education teacher(s) working in close collaboration with classroom teachers. They have a master’s degree and thereby the qualiﬁcations and skills to assess reading skills and provide intensified support. If the learning difficulties persist despite the support in school, a decision-making team comprising administrators, teachers, school psychologists, and guardians can decide to refer the child to further assessments outside the school. This multi-tiered framework resembles the Response to Intervention model (Fletcher & Vaughn, 2009). It was used in Finland in the late 1980s when the oldest participants of this study were assessed and has been officially implemented since 2010.

We endeavored to map and describe the experiences that have helped adult Finnish individuals with RD live and grow up with their disability. Furthermore, we included in this study only participants who did not show major attentional or emotional problems in childhood (based on guardians or teacher ratings in *ASEBA*; Achenbach & Rescorla, 2001) and who did not have other major learning disabilities (i.e., mathematics disability or comorbid RD and mathematics disability; *z*-score > −1.5 *SD*). This way we were able to reduce sample heterogeneity in terms of childhood comorbid problems which allowed us to draw conclusions on experiences of protective factors specifically related to childhood RD without other major disabilities that might confuse the findings. It should be noted that reading speed was used to measure reading skills and to define RD in childhood because Finnish orthography has consistent letter–sound correspondences, and reading accuracy is typically learned within the first few months in school (M. Aro & Wimmer, 2003), and therefore reading speed, rather than accuracy, is a better marker of RD.

We adopted a qualitative approach, as it allowed participants’ voices to be heard by giving them the opportunity to express their memories and thoughts about their experiences of protective factors. We see that knowledge on the lived experiences of individuals with RD can guide us in enshrining future generations from the discouraging impacts of RD by helping us identify the factors that can build resilience and sustain lifelong psychological well-being. This knowledge should guide planning and provision of appropriate support.

## Method

### Participants

This research is part of a larger longitudinal project tracing the lives of a group of individuals with learning disabilities (LDs, i.e., RD, mathematics disabilities or comorbid RD and mathematics disability), who attended the Clinic for Learning Disorders (CLD) in the city of Jyväskylä in childhood. This study’s participants were selected (see criteria below) from the CLD archival data. They had attended the clinic at 8–13 years of age (*M* = 10.7). All the participants were native speakers of Finnish, and they all came from the area of Central Finland. The distribution of the educational level of their mothers was comparable to the Finnish population in a scale ranging from (1) comprehensive school to (5) university degree (*M* = 3.00; *SD* = 1.22; range: 1–5).

The CLD offers neuropsychological assessment and counseling for children with LDs or problems related to attention. Children with social–emotional issues as their primary problem or who have global developmental delay are not referred to the clinic. Before referring a child to the CLD, his or her LD has been noticed in school and assessed by the special education teacher/school psychologist, and individually planned or intensified educational support has been provided. If the difficulties persist despite the provision of intensified support, the decision-making team referred the child to the CLD.

The participants were selected from the archives of the CLD based on two criteria: RD was their only childhood LD (i.e., their reading test *z*-score was ≤ −1.5), and they were above 20 years of age. Thus, individuals with comorbid mathematics difficulties (i.e., their mathematics test *z*-score was ≤ −1.5) or emotional and attention problems (*z*-score ≤ −1.0 in teacher/guardian ratings) in childhood were excluded to create a homogeneous group as much as possible in terms of the participants’ RD.

Written informed consent was obtained from all participants. The parents had given informed consent to use their children’s assessment data for research purposes when the participants had been assessed as children. Ethical approval was obtained from the University of Jyväskylä Ethical Committee, and the institutional consent to use the data was provided by Niilo Mäki Institute. The study was not preregistered.

Altogether, 76 individuals were identified with RD, based on the above criteria. No contact information was found for nine individuals as it had changed since childhood, and one person was deceased. Of these, 49 individuals (74.2% of the 66 reached individuals) agreed to participate in the follow-up assessments, including the interview conducted by licensed psychologists. The interviews were audio-recorded and transcribed. One individual participated only in the cognitive and academic assessment and thus had no interview data. This decreased the final sample size to 48, broken down into 30 (62.5%) males and 18 females (37.5%). The individuals who were included in this study in adulthood and those who did not participate in the adult-age assessments (*n* = 27) did not differ significantly in age or gender distribution, although there were slightly more males among the non-participants (22/27, 81.5%) than participants (30/48; 62.5%). No significant differences were found in their childhood RD level, verbal intelligence quotients (VIQ) or performance intelligence quotients (PIQ), emotional and attention problems, or in parental education level.

The participants had a mean age of 26 (*SD* = 4.7; range: 20–39) years. However, 10 (20.8%) participants were students, 27 (56.3%) had full-time or part-time jobs, 10 (20.8%) were unemployed/seeking a job, and 1 (2.1%) was completing his military service. Moreover, 3 (6.3%) did not pursue further studies after compulsory education, 33 (68.8%) had finished or were completing vocational school, 3 (6.3%) had finished or were completing high school, 6 (12.5%) had finished or were completing polytechnic studies, and 3 (6.3%) had finished or were completing studies in a university. The mean of the participants’ childhood Full-Scale IQ (WISC-R or WISC III; Wechsler, 1974, 1991) was 94.5 (*SD* = 8.1; range: 79–109), and the mean of their adult-age IQ (WASI IV; Wechsler, 2008) was 86.1 (*SD* = 15.3; range: 49–112). Their adult-age reading levels were on average 2–3 in a normed test battery for reading and spelling, with the maximum level being 9 (Nevala et al., 2006). The test battery (Nevala et al., 2006) used is the only test for adult reading skills available. It is standardized with comprehensive school ninth graders (*n* = 208). In the word reading task, the participant is asked to read aloud 30 words; in the pseudoword reading task, they are asked to read aloud 30 pseudowords; and in the text reading task, they read aloud a text for 3 min. In all tasks, the participants are asked to read as quickly and accurately as possible. In the word reading task, the mean level of the participants was 2.08 (*SD* = 1.44); in pseudoword reading task, the mean level was 3.40 (*SD* = 2.74); and in text reading task, the mean level was 2.27 (*SD* = 1.46).

### Procedure and Data Analysis

The semi-structured interview consisted of questions tapping into four domains: (a) experiences of social relationships and support, (b) areas of life affected by RD, (c) personal strengths, and (d) future goals and aspirations. The interviewees expressed experienced protective factors through the interview, and all expressions related to protective factors were included in the analysis. Each interview lasted about 40–60 min, and they were conducted by licensed psychologists. The data were treated anonymously and analyzed with qualitative inductive content analysis, which aims to identify and describe “a condensed and broad description of the phenomenon” (Elo & Kyngäs, 2008), without pre-decided theoretical perspectives (Sarajärvi & Tuomi, 2018, p. 108). According to the work by Kyngäs (2019, p. 13), “the ultimate aim is to produce abstracts of the raw data that summarize the main categories, concepts and themes, and provide indications of potential theoretical relationships.”

The data analysis started with familiarization with the data by reading and re-reading of the transcribed interview data with an intention to gain an overall understanding of the phenomenon. Similarly, data were reduced by filtering out the material that was irrelevant to the research question. While reading the interview transcripts, the aim was to identify analytical meaning units that could be grouped into larger entities and subcategories. Here, words, sentences, and chunks of text that contain the same meaning were regarded as analytical meaning units, which were further condensed into “condensed meaning units” (Erlingsson & Brysiewicz, 2017). The *condensation* is a process where while shortening the data, the core meaning is preserved (Graneheim & Lundman, 2004), and which results condensed meaning units that are shortened versions “of the same text that still conveys the essential message of the meaning unit” (Erlingsson & Brysiewicz, 2017). Next, based on the similarities and the differences, the condensed meaning units were grouped into subcategories and again into main categories (i.e., clustering phase).

For example, the condensed meaning units “The family’s supportive attitude and belief in the chances of progressing in school” and “Close relationships and talking about things with a parent” had the same overall meaning and formed a subcategory “Emotional support from family.” Similarly, the condensed meaning units such as “Reading aloud with the family” and “Older siblings’ help in homework” had the same overall meaning and formed a subcategory called “Help of the family in homework.” Then, the subcategories “Emotional support from family” and “Help of the family in homework,” which were seen to belong together, were further grouped with other similar subcategories to form a main category “Supportive family.” The central idea was to identify as independent and mutually exclusive categories as possible (Graneheim & Lundman, 2004). Finally, the main categories with the same thread of an underlying meaning or latent content were grouped together to form a subtheme and then, related subthemes to form a theme (Graneheim & Lundman, 2004).

To guarantee loyalty to the data, the transcripts were read through several times during the analysis. The trustworthiness of the findings was further ensured with the practices of communicative validity where “valid knowledge emerges as conflicting knowledge claims are argued in a dialogue” (Kvale, 1996, p. 6). Although one person reduced the data and identified the meaning units, all further steps were taken collectively by debating and testing the ideas in a dialogue. All authors, for instance, discussed and made sure that the condensed meaning units genuinely captured the core meaning of the original data. Throughout the process of categorizing, the authors provided and argued probable and alternative ways of interpreting the data and through a dialogue reached a consensus. The group of researchers served as a space for reflexive processes, where the members as “critical friends” ensured the examination and identification if and how researcher’s “assumptions and views might affect her interpretation of the respondent’s words (Mauthner & Doucet, 2003, p. 419).”

## Results

The analysis yielded three main themes: (a) *Factors Supporting Personal Development*, (b) *Factors Supporting Social Well-being and Belonging*, and (c) *Factors Supporting Learning*. Each theme included two to four subthemes, each with two to six main categories comprising several subcategories, as shown in Tables 1–3. In the following subsections, the themes, subthemes, main categories, and subcategories are introduced with selected citations (Cit) of the interviews.

**Table 1.** Theme 1: “Factors Supporting Personal Development,” Its Subthemes, Main Categories, and Subcategories.

Subtheme	Main category	Subcategory
Personal attributes supporting learning	Positive attitudes towards learning	• Diligence
		• Persistence
		• Resilience
		• Willingness to learn
		• Belief that obstacles can be overcome
		• Competitiveness
		• No comparison to others
	Learning skills and strategies	• Finding own ways of learning by experimentation and trying
		• Pacing
		• Routines
		• Ability to be analytic and learn from one’s mistakes
		• Ability to focus attention
		• Ability to use visual and auditory memory
Accepting RD	Personal acceptance	• Self-esteem
		• Self-understanding
		• Positive attitude: “does not matter”
		• Personal strengths
		• Understanding what enough is /sense of proportion
		• Knowing that I am not the only one with RD
	RD recognition by others	• Awareness and understanding of RD by adults at school
		• Diagnosis helps to understand RD
		• Understanding of the RD in the family
Agency	Participation in decision making	• Responsibility of one’s own choices and actions
		• Participating in meetings and decision-making concerning one
		• Possibility to influence in educational arrangements
	Proactivity towards learning and help-seeking	• Being initiative • Asking for help
Finding one’s own niche	Self-actualization	• Finding areas where is good
		• Finding one own career choice
		• Meaningful and helpful/beneficial hobbies
	Finding befitting educational tracks/careers	• Finding career, where RD does not matter • Proceeding in career without formal schooling

*Note*. RD = reading disability.

### Theme 1: Factors Supporting Personal Development

The first theme encompassed experiences related to the interviewees themselves, that is, their personal attributes supporting learning (Subtheme 1), accepting RD (Subtheme 2), gaining agency (Subtheme 3), and finding one’s own niche (Subtheme 4) (see Table 1).

#### Subtheme 1: Personal Attributes Supporting Learning

The interviewees brought up several personal attributes that they had experienced as supporting learning despite RD. “Positive attitudes toward learning” such as diligence, persistence, patience, and resilience helped them cope with RD despite the arduousness and their slowness in learning and doing homework. Emotional drain caused by RD was in fact sometimes experienced as something that built their persistence and resilience (Cit 1), increased their grit and willingness to succeed, and strengthened their belief in being able to overcome their difficulties (Cit 2):

Of course, I had to work twice as hard as the others . . . and there’s the fact that it may have built my character so that I can manage to do it a bit longer and not give up as easily. (Cit 1)
Challenges can be overcome . . . you learn to fix your own mistakes, you can achieve things even if you’re a bad reader or make mistakes, and you can develop and challenge yourself. (Cit 2)

Furthermore, the respondents discussed about the importance of different “Learning skills and strategies.” Learning practices such as finding their own ways of learning, routines, and pacing and timing studies according to one’s own resources, learning through experimentation, and the abilities to be analytical and learn from one’s mistakes, to focus attention, and to use visual and auditory memory (multichannel) were perceived as helpful skills and strategies (Cit 3):

I’ve found that I learn and remember best when I read and write it down and say it out loud and then go through it again, so that I get the same info through many channels. (Cit 3)

#### Subtheme 2: Accepting RD

The interviewees emphasized the importance of accepting and recognizing RD, both by themselves and by others. “Personal acceptance” constituted factors, such as having good self-esteem and self-understanding despite RD (Cit 4), and adjustment to and acceptance of RD (Cit, 5), which were regarded as buffering against difficulties. Although their RD was experienced as harmful during their school years, some described how they had learned to be more accepting and to have a sense of humor about their RD over the years and to put it in perspective, and they realized that others also had RD (Cit 6):

I’ve always had a really high self-esteem . . . I’ve been, like, I have this problem and it is what it is, but I don’t feel like it has in any way brought me down. (Cit 4)
If the content is right and the grade is satisfactory for me, or good, somehow I can’t be bothered to worry too much, because I know that it will get better up to a certain point, I’ll probably never be a top writer, but it doesn’t really matter. (Cit 5)
There are others like me, and there are even princesses with dyslexia. (Cit 6)

In addition to their own acceptance of the difficulties, “RD recognition by others” was considered important. Especially, the awareness, willingness to help, and understanding expressed by the adults at the school were relevant. The teachers showed interest and referred the students to special education/psychologist for an assessment (Cit 7). Awareness and acceptance expressed by the teachers were viewed as also relevant for the classroom climate and transmission of information. Being diagnosed or having RD assessed (Cit 8) and talking about RD at home (Cit 9) and in school were experienced as informative and consoling:

My primary school teacher . . . sent me off to be tested in all kinds of ways and . . . they were interested in helping me. (Cit 7)
When we found out what it was, it was comforting to know that I’m not a lost cause. (Cit 8)
They figured things out and . . . the reading difficulties were also talked about in general at home, and it was made clear pretty quickly that it wasn’t that I was stupid or anything. (Cit 9)

#### Subtheme 3: Agency

Agency as a supportive factor constitutes of the experiences related to the joint decision-making and proactivity. “Participation in decision-making” constituted of factors, such as taking responsibility for one’s own decisions and actions, attending meetings, taking part in the decision-making process, and being able to influence pedagogical arrangements and solutions, was perceived as supportive (Cit 10):

A kind of responsibility that I’ve learned to take for my own actions, always in relation to my life situation . . . I’ve been able to make my own choices which have luckily turned out to be good ones. (Cit 10)

“Proactivity toward learning and seeking help” covers such experienced protective factors as the interviewees’ own proactivity in terms of education and learning. Their experiences of being active in seeking help (Cit 11) and having initiative in terms of learning (e.g., actively searching for information and doing things that support learning) were described by the interviewees.

I can make a difference [in getting help] if I need it, I’ll ask for support or help. (Cit 11)

#### Subtheme 4: Finding One’s Own Niche

The subtheme combines the protective factors related to fulfilling one’s potential and finding suitable education. “Self-actualization” covers experiences about the relevance of finding one’s own strengths, educational tracks/careers with minor requirements for reading and writing, and environments that felt like one’s own and suited one’s characteristics. The participants talked about the finding areas of competence, interest, and success (Cit 12), which were thought as stabilizing and counterbalancing the difficulties experienced because of RD:

It’s really important to have experiences of accomplishment and to find your own thing, you don’t have to be good at everything but it’s about finding your own thing. (Cit 12)

“Finding befitting educational tracks/careers” such as finding appropriate educational tracks and making suitable career choices with minor requirements for reading and writing were experienced as motivating and encouraging the participants to invest in studies (Cit 13). Similarly, choosing lines of business and realizing the possibilities to proceed in their careers without needing to study further reduced their worries (Cit 14).

The study subjects are much more interesting to yourself, so you tend to put more time into it and you feel that it’s more useful . . . especially, when you’re like studying for your own profession, it feels even more tangible. (Cit 13)
I’ve finished my schooling . . . so I’m not constantly worrying about school and that learning difficulty anymore . . . it’s not like I need to learn new things every day nowadays. (Cit 14)

### Theme 2: Factors Supporting Social Well-Being and Belonging

The second theme covered experiences related to social relationships and social systems providing experiences of protective factors. It included two subthemes, namely “Supportive Social Relationships” and “Belonging to School” (see Table 2).

**Table 2.** Theme 2: “Factors Supporting Social Well-being and Belonging,” Its Subthemes, Main Categories, and Subcategories.

Subtheme	Main category	Subcategory
Supportive relationships	Supportive family	• Emotional support from family
		• Presence of parents, devotion of their time and attention
		• Help of the family in homework
		• Parents’ activity in search for help
		• Resources
		• Help of the close ones in adult-age
	Supportive others	• Peers’ support and help
		• Support and help from adults in the school
		• Support and help from colleagues
Belonging to school	Sense of community	• Sense of cohesion in the classroom
		• Peer support in the special education small group
		• Culture of open dialogue
	Psychological safety in school	• Caring
		• No tolerance for bullying
		• Accepting atmosphere in the school

#### Subtheme 1: Supportive Social Relationships

The interviewees described supportive relationships, both in the immediate family and other close relatives, and with persons in school and the workplace. A “supportive family” was regarded as providing emotional support, dedicating time and attention, and helping in schoolwork (Cit 15). The interviewees highlighted the importance of parents’ persistence in and devotion to seeking help and providing possibilities for hobbies. In adulthood, support and help, for instance, in the form of proofreading, were provided by one’s partner/spouse or significant others:

I write, sometimes summaries and sometimes essays . . . my mother gives me the topics . . . we think about questions . . . and she gives me feedback on them, and then I go through them with her and think about what could be improved. (Cit 15)

“Supportive others” were those from outside the immediate family: peers, colleagues, and adults in school. Peers provided concrete and reciprocal help in schoolwork, caring and support, and counterbalance for schoolwork. Closeness to caring, supportive, and respectful adults in school was also experienced as important (Cit 16). The support by these adults helped the participants to be persistent and keep trying (Cit 17). In a similar vein, appreciative colleagues at work were experienced as sources of support when RD could be openly discussed and there were others with similar difficulties:

It [seeing the special education teacher] was almost like the highlight of the week . . . I really liked her a lot . . . they were someone who kept believing in me. (Cit 16)
The classroom teacher’s contribution was quite big . . . it did help, and then there’s the fact that you kept on trying, and it does help improving if you practice, you can’t help but improve. (Cit 17)

#### Subtheme 2: Belonging to School

In addition to relationships with important individuals, the interviewees also described the relevance of a more general ambience that had given them a sense of coherence and belonging, and made them feel safe in school or in a small, special education group. A “Sense of community” was experienced in the feeling of cohesion and in interconnectedness in the class, in peer support in a small educational group, and in a classroom culture of open dialogue. Some interviewees experienced small groups as having a good team spirit (Cit 18). In contrast, referring to the same topic, some interviewees encountered a lack of cohesion when they left their own class to join a small group, which made them feel like outsiders and diminished their self-esteem. Having classmates with LDs also made it feel easy to openly share their RD (Cit 19):

We had our own cool group where we went, and they thought it was so cool . . . we were maybe like more okay than the rest (laugh) when we were there. (Cit 18)
There were other people with learning difficulties in our class, so the issue was in a way like out in the open. (Cit 19)

“Psychological safety” comprised such protective experiences as a school climate with a caring and accepting stance and zero tolerance for bullying (Cit 20); the same was expressed by some interviewees through their negative experiences of being bullied or feeling unsafe. The accepting stance was experienced, for example, in the teacher’s discretion in reading situations and in the caring expressed by the teacher (Cit 21):

I was part of the group and there was no taunting there. (Cit 20)
The special education teacher . . . asked me how it went and how I’d been coping. (Cit 21)

### Theme 3: Factors Supporting Learning

The third theme covered practical solutions and pedagogical arrangements that had helped the interviewees in learning. They described some practices, solutions, and arrangements (see Table 3).

**Table 3.** Theme 3: “Factors Supporting Learning,” Its Subthemes, Main Categories, and Subcategories.

Subtheme	Main category	Subcategory
Flexible and individually tailored practices	Appropriate support for learning	Rightly focused and rightly timed support
		• Regular/systematic special education
		• Individual/ face-to-face support from the special education teacher
	Flexible pedagogical practices	• Starting school later/postponing school start
		• Flexibly changing between educational small group and mainstream classroom
		• Flexibly changing between theoretical and practical studies
	Individualized teaching arrangements	• Teacher in the educational small group had time
		• Educational small group allowed for asking questions
		• Rightly set goals and requirements
		• Good pace
		• Less pressure in the educational small group
	General pedagogical techniques	• Learning by playing
		• Learning by doing
		• Acquiring strategies and tools of learning
		• Repeating in teaching
	Digital and technological solutions	• Audiobook
		• Using computer for writing
		• Computerized exercises
	Teaching and acquiring metacognitive skills	• Learning strategic studying skillsLearning to concentrate
Learning outside school	Everyday learning outside school	• Responding to challenges requiring learning in daily life
		• Learning though hobbies
	Neuropsychological rehabilitation outside school	• Neuropsychological intervention for reading

#### Subtheme 1: Flexible and Individually Tailored Practices

The interviewees talked about several practices that had been experienced as supportive. They explained that appropriately focused support, flexibility and individualization, innovative and practical teaching techniques, use of digitalized solutions, and improved skills had helped in their learning. “Appropriate support for learning” constituted protective factors, such as individually tailored, rightly focused, timely, and regular/systematic support, which also considered future needs (Cit 22; describing not so appropriate support: Cit 23). Moreover, the face-to-face support provided by the teacher was experienced as less stressful (Cit 24) than classroom instruction:

Well it’s that they come right next to you and then, I don’t know, they like concentrate on you so that you are sure to understand it. (Cit 22)
Together we thought about which field to go into, but it could have been more extensive . . . like if I go to some vocational school, what kind of accommodation they do there, they didn’t know that much about it, if it would have been possible . . . maybe more information about how to cope when I’m an adult. (Cit 23)
When it was just the two of us there, I didn’t need to worry about it being a little slower, the reading, or making mistakes or stopping, it was no problem. (Cit 24)

“Flexible pedagogical practices,” such as pedagogical solutions tailored according to the students’ needs, such as starting schooling later than others, switching between the mainstream classroom and a small educational group, and alternating between theoretical and practical studies (Cit 25):

Well, doing the practical things, and I only had to sit in the classroom for periods of a few of weeks, and even that was training things in practice. (Cit 25)

“Individualized teaching arrangements,” such as teachers having the time for individual support (Cit 26), allowing space for questioning and thorough teaching that fostered a profound understanding (Cit 27), which often took place in small, special education groups were experienced as protective factors. In these small groups, the interviewees experienced less pressure, learning at a good pace, and appropriate requirements (Cit 28). Good support for learning also included some pedagogical techniques or tactics:

Individually, yes, they gave advice by showing me how to do it from a book and taught me by sitting next to me like my mother always taught me too. (Cit 26)
We went through the things as thoroughly as possible so that that I was sure to understand it. (Cit 27)
They made sure that it wasn’t too easy, but that you could manage, it was like thinking about the future. (Cit 28)

“General pedagogical techniques” that were experienced as helpful included techniques, such as repetition, learning by doing, or learning by playing (Cit 29):

For me it’s best if someone can show me how to do it. (Cit 29)

“Digital and technological solutions,” such as, use of audiobooks and computers (Cit 30) were experienced as useful and helpful for learning:

When they [the teachers] brought in that computer stuff, like teaching stuff that you could use to practice, maybe that’s where this interest in computers came from. (Cit 30)

“Teaching and acquiring metacognitive learning skills,” such as teaching and being trained in useful learning methods (Cit 31), understanding the meaning of concentration to achieve a learning outcome (Cit 32), and learning to concentrate were experienced as protective skills:

When I was young . . . I think I got some tools for learning which are useful for learning better. (Cit 31)
. . . everything is based on whether you concentrate or not. (Cit 32)

#### Subtheme2: Learning Outside School

“Everyday learning outside school,” that is, situations confronted in daily life at work or in social situations challenged interviewees to learn new skills, like a new language (Cit 33) or social skills and courage (Cit 34). Others also mentioned learning through hobbies (e.g., learning English by playing computer games):

Working life has kind of challenged me or pushed me to learn languages [. . .] like the prognosis that the kid had . . . or they have thought that the guy reads slowly, and his language learning looks really bad. . . I kind of break all those predictions. (Cit 33)
I was a bit shy and didn’t even dare to open my mouth . . . going to work in a shop taught me quite a lot . . . there were so many people there that I’m not that shy anymore, and I do open my mouth, maybe even a bit too much nowadays. (Cit 34)

Only one participant had “Neuropsychological rehabilitation outside school.”

## Discussion

Using semi-structured interviews and qualitative content analysis, we aimed to map experienced protective factors of adults diagnosed in childhood with RD. The identified three themes covered protective factors that were related to personal attributes and development of the interviewees, their relationships with others and their communities, and actions and arrangements in their environments, mainly in school. The themes were named as follows: (a) *Factors Supporting Personal Development*, (b) *Factors Supporting Social Well-being and Belonging*, and (c) *Factors Supporting Learning*. The present findings are in line with the few existing studies which have indicated such factors as personal characteristics, early diagnosis/recognition, caring and understanding environment, and finding a niche as supportive (Doikou-Avlidou, 2015; Hellendoorn & Ruijssenaars, 2000; Lithari, 2019; McNulty, 2003; Stampoltzis & Polychronopoulou, 2009). However, our findings revealed protective elements that not commonly reported, such as, agency, belonging to the school and the school’s acceptive and respectful atmosphere, and a variety of helpful pedagogical practices, and RD experienced as something that could also strengthen positive attributes and resilience. The sources of protective factors brought up by the interviewees underline the relevance of the environmental and social protective factors along with the personal characteristics of the individual, and they can guide future pedagogical approaches and intervention development.

### Factors Supporting Personal Development

The theme *Factors Supporting Personal Development* comprised four subthemes and included personal attributes experienced as protective, acceptance of RD (by oneself and by others), one’s own agency (participation; proactivity), and experiences of finding one’s own niche. The interviewees indicated several attitudes (e.g., patience, willingness to learn, persistence) and skills and strategies (e.g., having routines and being able to focus) that helped them cope with their RD. In a broad sense, the core substance of the theme constituted coping with the disability, accepting it, and learning to know one’s strengths. This notion is in line with Hellendoorn and Ruijssenaars’ (2000) suggestion that knowing one’s own abilities and making good use of them is important for the adjustment of individuals with RD.

The interviewees also noted how having RD strengthened their positive attributes or was the source of the latter’s emergence. This is consistent with the recent findings of Shaywitz et al. (2020), who reported that university students spoke about being more determined or hardworking because of their dyslexia. Likewise, Nalavany et al. (2011), who studied the psychosocial experiences of adults with RD, found that many of their participants had successfully compensated for their RD through perseverance and a variety of adaptive coping skills. Accordingly, Givon and Court (2010) have concluded that determination is a relevant adaptive coping strategy, as it features determination to face and deal with the challenges, and to display one’s personal competencies despite the learning disability. Thus, personal characteristics and skills related to building grit and resilience despite RD—or because of it—appear to be important protective factors. The attitudes of being persistent and not giving up might build resilience (see Tanner, 2009; Terras et al., 2009) and buffer against emotional distress and low self-esteem (e.g., Lithari, 2019; Riddick et al., 1999).

The second subtheme under the *Factors Supporting Personal Development* theme was acceptance. Our participants unequivocally stressed that acceptance of the difficulties and recognition of RD by oneself and by others were important and experienced as buffering against a negative self-concept and low self-esteem. Along the same lines, recognition of the difficulties and obtaining a diagnosis have been identified in some earlier studies as important for coping and for positive identity development (Hellendoorn & Ruijssenaars, 2000; Lithari, 2019; Riddick et al., 1997; Spekman et al., 1992; Stampoltzis & Polychronopoulou, 2009). Similarly, in their seminal work among individuals with learning disabilities, Goldberg et al. (2003) noted that compartmentalization was the key self-awareness component related to success in their studies and in life. However, our data suggested that acceptance is more than diagnosis or recognition of the disability, as it may include recognition of one’s own strengths, having good self-esteem and gaining self-understanding and adjustment.

The third subtheme under the *Factors Supporting Personal Development* theme was agency. Having experiences of participation and being proactive were mentioned as protective experiences. In previous studies, this theme has rarely been discussed (see however Burden, 2008; Goldberg et al., 2003; Raskind et al., 1999). This is rather surprising because agency, characterized by intentionality, forethought, self-reactiveness, and self-reflectiveness, enables individuals to act (Bandura et al., 2001), and it is also related to self-determination (Ryan & Deci, 2000) and self-regulated learning (Winne, 2018).

The fourth subtheme under the *Factors Supporting Personal Development* theme was niche. In his study among adults diagnosed with dyslexia, McNulty (2003) noticed that having a niche in late adolescence and young adulthood improved adaptation and self-esteem and could dramatically change a person’s life. Correspondingly, our participants emphasized the importance of finding meaningfulness in activities and career choices (cf. self-actualization) and discovering suitable educational tracks. This raises the question of how a school and its teachers and other personnel can support students with RD in finding their paths and back them up in the process, which probably includes setbacks and disappointments, and it is not as straightforward as one would hope.

### Factors Supporting Social Well-Being and Belonging

The theme *Factors Supporting Social Well-being and Belonging* comprised two subthemes: supportive relationships and sense of belonging to school. The support of the significant others in the family, as mentioned by our interviewees, has been recognized in several earlier studies as a crucial protective element in the lives of individuals with RD (e.g., Glazzard, 2010; Hellendoorn & Ruijssenaars, 2000; Nalavany & Carawan, 2012; Nalavany et al., 2011; Stampoltzis & Polychronopoulou, 2009). The relevance of the family was also shown by Nevill and Forsey (2023) in their systematic review of the qualitative research on the primary and the secondary education of students with dyslexia. They concluded that such students would face more disabling barriers if it were not for the advocacy of their mothers.

The findings suggest that due to their key role, parents of children with RD might also benefit from support services. Therefore, future research should include the voices of parents and significant others to increase the understanding of their experiences and perspectives (see Hellendoorn & Ruijssenaars, 2000; Nalavany & Carawan, 2012). Recently, Wilmot et al. (2023) interviewed children with RD and their mothers, and their findings emphasized the importance of the whole community and family–school connections for psychosocial well-being. Studies among guardians of children with RD could also prove a broader perspective on sociocultural barriers to learning experiences of children with RD (Leitão et al., 2017).

Our interviewees brought up the relevance of a supportive school environment, that is, the support provided by teachers, peers, friends, and colleagues, as did the participants of some earlier studies (Doikou-Avlidou, 2015; Leitão et al., 2017; Nalavany et al., 2011). The supportive others played a vital role in cultivating emotional reactions to RD and building grit, which can have a positive effect on self-esteem and psychological well-being. This concords with Ross’s (2021) conclusion stressing that young people’s conceptualization of their RD reinforced their interactions with others and was influenced by those interactions.

The data showed that in addition to support received from significant others, classmates, and school personnel, the school atmosphere can be experienced as an important protective factor. The sense of community and psychological safety experienced in school or in a small group created a sense of belonging to school. As noted in the work by Hellendoorn and Ruijssenaars (2000), positive school experiences are relevant for accepting one’s RD, which together with our findings, stress the teachers’ and the whole school community’s role in promoting a welcoming atmosphere and attitude among peers and school personnel; a similar acceptance should be expressed in workplaces. At best, feeling psychologically safe allows for exercising one’s agency to engage in new experiences and interactions, as there is no reason to fear rejection, embarrassment, or punishment, and thereby, it also supports learning.

Overall, the two above themes underline the significance of genuine acceptance and recognition of RD and seeing the person instead of the disability at home and in the school community. The ethos of acceptance and support allows students’ active participation in decision-making and their proactivity, which can then increase their motivation for learning and support them in finding their own educational path and niche.

### Factors Supporting Learning

The interviewees mentioned the importance of individual and flexible educational support in school, and some also brought up learning possibilities outside school. Several diverse practices were also mentioned, which are reflected in the number of main categories (six) under the subtheme “Flexible and individually tailored practices.” The multitude of protective experiences related to these practicalities underlines the value of trying to find individually suitable and best-fitting methods and environments for students with RD. This finding also highlights the key role played by the school personnel in helping people with RD although their effort often does not show obvious results immediately. Besides, the data showed that engaging in learning processes outside can provide informal learning environments, and at best, they can enhance the individuals’ motivation to learn and build self-efficacy through daily mastery experiences.

### Strengths and Limitations

As with all research, this study has limitations that should be considered when interpreting the results. First, relying on participants’ self-reporting poses a potential risk of bias, and thus, the nature of our data and the sample (e.g., Finnish adults, whose RD was assessed in childhood) should be kept in mind when interpreting the results. It is likely that the experiences are not similar in all contexts and all age groups as educational systems and views on learning disabilities change in time and between countries/communities. To avoid the potential bias related to retrospective self-reporting (e.g., altered memories), in future studies, mixed-method approach combining data both on subjective experiences and more objective data on environmental factors is recommended. In addition, although adults may be able to better reflect on their experiences, similar interviews could be conducted among adolescent currently in school experiencing the support services. Our study was descriptive and inductive, and its purpose was to understand the interviewees’ lifelong experiences, and we do not claim to suggest causal processes. Rather, the findings can feed into future research that seeks to gain a better understanding of promotive and protective factors for people with RD or to develop interventions.

Second, the sample size (48 interviewees) for this type of in-depth qualitative data collection can be regarded as large and therefore a strength. However, it is not certain to what extent their experiences are representative of a larger population with RD or individuals with RD in other cultural contexts, especially as the age range was somewhat limited and not all individuals could be reached, and no information is available for the reasons their contact information was not detectable and the possible bias caused by this cannot be inferred. There was an overrepresentation of males which is consistent with diagnosis rates and can thus be considered a strength; otherwise, the sample was representative of the Finnish population attending special education due to RD (i.e., maternal education, age at the time of detecting RD). The participants were all former clients of the CLD, their RD was rather severe and persistent (see Eloranta et al., 2019), and some of their school experiences date back to the 1980s and the 1990s. Therefore, their experiences may differ from those of their counterparts receiving special education support today or in other cultural contexts, especially if special educational support is not as easily available as in Finland. However, the special education support provided in their school years does not differ much from the one currently provided in Finland; it does not require diagnosis but is based on educational needs, and it is provided by special education teacher with a master’s degree. Still, more research in other cultural and educational contexts is needed.

Additionally, the participants did not have evident co-occurring mathematics disability, emotional problems, or attention-deficit/hyperactivity disorder (ADHD) in childhood, all of which are quite often recorded in the RD population. Due to narrowing down the sample to individuals with a rather specific RD, we can conclude that other childhood difficulties did not affect our findings and our findings concern experiences of individuals with RD. At the same time, it must be recognized that the findings may be different among RD individuals with co-occurring problems which constrain the generalizability and validity of the findings to a broader population with RD. Furthermore, all interviewees volunteered to participate, and it can only be speculated whether those who declined to participate would have had different types of experiences.

### Implications for Practice and Research

This study’s findings indicated that along with the personal attributes of individuals with RD, several sources of protective factors can be identified from the social environment and social relationships, and that individual and flexible support, and the school atmosphere, can be experienced as protective factors. With the awareness of the protective elements for success comes the ability of the professionals to offer research-informed practices and to assist individuals with RD in approaching their lives from the perspective of their varying strengths. The present findings especially denote the relevance of the attitudes and practical actions taken by the pedagogical professionals and peers—they can make a difference. Thus, teachers and other people close to individuals with RD should be encouraged to identify the best-suited ways to support the latter as these endeavors can bear fruit afterwards, and their superiors and the community should support them in this work.

Our data suggested several targets of support. First, providing experiences to develop such characteristics as perseverance, determination, and adaptive coping skills should be embedded in all educational support. In addition to training students with RD in the reading skill itself, it is important to provide support for positive identity development and self-knowledge during the school years, and beyond. Second, the findings underscore the importance of the pedagogical personnel’s awareness and cognizance of the signs of RD and their own ability to guide the student and the family to assessment and steer them toward understanding the difficulty and its implications for both learning and identity development. Recognition and acceptance of students’ difficulties is the first requirement to enable their own acceptance of the difficulties, which is necessary for them to be able to identify their needs and make the best of their strengths in their quest for success and future accomplishments. Only through genuine acceptance of the RD can unquestionably supportive relationships and psychologically safe environment be formed.

Third, the findings imply that actively inviting students to participate in decision-making processes can increase students’ sense of agency and help them take their learning process and teaching arrangements in their own hands; thereby, it can reinforce their motivation for learning. Fourth, together with support and acceptance shown by the school community, individually tailored practical solutions with flexibility and possibilities to experiment with diverse subjects and learning methods during the school years can greatly promote development of healthy identity. Fifth, our findings indicate that support of significant others is needed, not only during childhood but also during the demanding life period of adolescence and adulthood, when the help provided by significant others outside the immediate family becomes even more relevant.

To gain a better understanding of the protective and the unhelpful—and the harmful—factors and processes affecting the lives of individuals with RD, future research should take a step away from the individual-focused models and integrate them with sociocultural models of disability (see Nevill & Forsey, 2023) recognizing the relevance of the community and the social nature of RD. Furthermore, mixed-method research approach combining interview with questionnaire or observational data is recommended. Future research should also use multiple sources of information to include the voices of significant others and the whole community in addition to the perspectives of individuals with RD.

Overall, our findings substantiate the recent views (e.g., Bazen et al., 2023) on the relevant influence of personal, socioemotional, and environmental factors in the formation of the consequences of RD. The results also confirm the importance of taking all these elements into account, in addition to RD or cognitive deficits, in further research and practices that will seek to understand RD, its long-term consequences, or develop interventions.

## Conclusion

Our findings demonstrated that several types of experiences of protective factors can be detected when using the voices of the individuals with RD as the source of information. The findings are in line with Catts and Petscher’s (2022) Cumulative Risk and Resilience Model of Dyslexia claiming that protective factors may be divided into those that are internal and external to the individual. They refer to internal resilience factors, such as “growth mindset,” “task-focused behavior,” and “adaptive coping strategies,” and to external resilience factors, such as “instruction” and “family and peer support.” In addition to these, our participants, perceived school as a relevant source of protective experiences for personal development and for social well-being and belonging. This indicates that school is not only a source of instruction but instead, school community can also be experienced as an important social community providing acceptance, sense of belonging, and support for personal development and agency.

Previous qualitative studies have demonstrated that, in addition to more severe academic and mental health problems, low self-esteem (e.g., Ingesson, 2007; Stampoltzis & Polychronopoulou, 2009), lack of academic choices and negative school experiences (e.g., Bacon & Bennett, 2013; Doikou-Avlidou, 2015), and problems in social relations (e.g., Doikou-Avlidou, 2015; Lithari, 2019) are common among individual with RD. Reflecting our results on experiences of protective factors against these earlier qualitative observations, it seems possible that these more subtle problems in social and psychological well-being during the life course and individually tailored instructional support can be relevant targets for support. That is, receiving support that protects from these adverse experiences may break the negative cycle which could otherwise lead to more severe problems in education, income, and mental health (T. Aro et al., 2019, 2024; Ghisi et al., 2016; Hakkarainen et al., 2015; Klassen et al., 2013; McLaughlin et al., 2014; Wilson et al., 2009). It is possible that with well-targeted support, the encountered difficulties related to RD may build resilience (see McNulty, 2003; Nalavany et al., 2011), at least among some individuals, which could partly explain the variation in long-term consequences.
